# Supplementary material for: Outstanding performance of an invasive alien tree Bischofia javanica relative to native tree species and implications for management of insular primary forests
Source: PeerJ. 2020 Jul 23;8:e9573. doi: 10.7717/peerj.9573 (PMC7382941; doi:10.7717/peerj.9573)
Supplement: Figure S1 — (a) Haha-jima Island and the Sekimon forests on uplifted limestone and (b) the census plots in the Sekimon forests. Crosshatched rectangles are the two 2-ha plots surveyed in the present study; the gray-shaded rectangle at the southern edge of the western plot represents the plot studied by Shimizu (1994), of which the southernmost part was lost to a landslide. the irregular gray-shaded area at the northeastern corner of the eastern plot represents a former Bischofia javanica plantation. Map credit: Abe, Tanaka & Shimizu (2018), Copyright ©2018, Springer Nature. [file peerj-08-9573-s001.pdf]

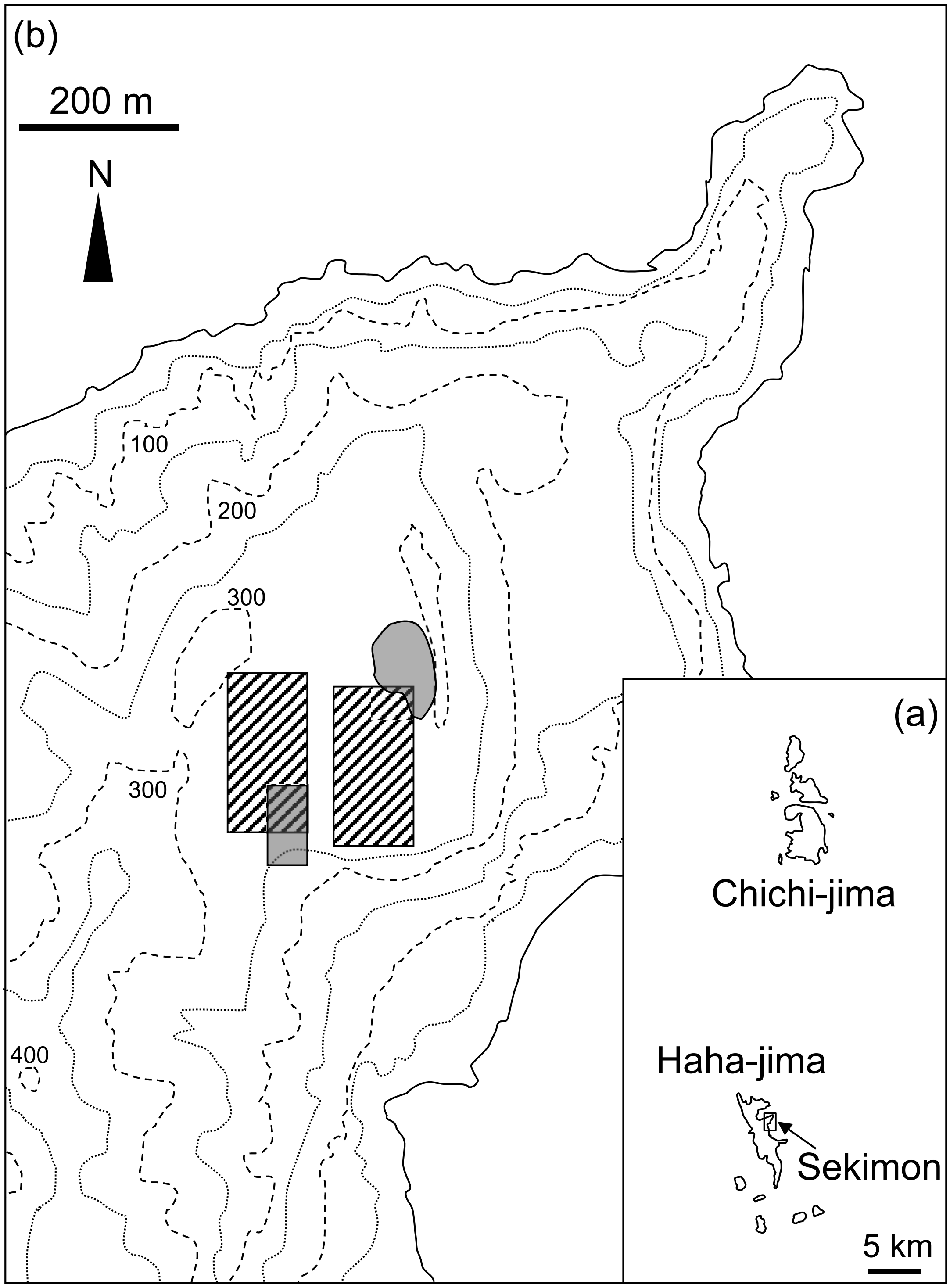

Fig. S1 Location of study site. (a) Haha-jima Island and the Sekimon forests on uplifted limestone and (b) the census plots in the Sekimon forests. Crosshatched rectangles are the two 2-ha plots surveyed in the present study; the gray-shaded rectangle at the southern edge of the western plot represents the plot studied by Shimizu (1994), of which the southernmost part was lost to a landslide. the irregular gray-shaded area at the northeastern corner of the eastern plot represents a former *Bischofia javanica* plantation.
