# Supplementary material for: Outstanding performance of an invasive alien tree Bischofia javanica relative to native tree species and implications for management of insular primary forests
Source: PeerJ. 2020 Jul 23;8:e9573. doi: 10.7717/peerj.9573 (PMC7382941; doi:10.7717/peerj.9573)
Supplement: Table S1 — The 1987 values are from the Shimizu (1994) and the 2006 values are collected by the present study within the former Shimizu plot (60 m × 50 m). Species are listed in descending order of BA in 1987. Numbers within parentheses after the BA values in 2006 represent the species order based on the BA in 2006. Only Bischofia javanica is an alien species. Species order is based on descending order of BA in 1987. [file peerj-08-9573-s002.pdf]

Table S1 Changes in the number of stems (N) and basal area (BA) of trees (DBH ≥ 10 cm) between 1987 and 2006. The 1987 values are from the Shimizu (1994) and the 2006 values are collected by the present study within the former Shimizu plot (60 m × 50 m). Species are listed in descending order of BA in 1987. Numbers within parentheses after the BA values in 2006 represent the species order based on the BA in 2006. Only *Bischofia javanica* is an alien species. Species order is based on descending order of BA in 1987.

	1987		2006	
Species	N (/ha)	BA (m^2/ha)	N (/ha)	BA (m^2/ha)
Pisonia umbellifera	187	36.3	250	35.5 (1)
Ardisia sieboldii	797	18.6	1120	24.7 (2)
Elaeocarpus photiniifolius	63	12.0	113	18.2 (3)
Planchonella obovata var. *obovata*	30	4.4	17	1.1 (9)
Hibiscus glaber	20	3.8	37	4.1 (4)
Melia azedarach	10	2.5	13	3.7 (5)
Cyathea mertensiana	50	1.5	30	1.2 (8)
Psychotria homalosperma	30	1.3	17	0.3 (11)
Bischofia javanica	17	0.9	47	2.5 (6)
Zanthoxylum ailanthoides var. *inerme*	10	0.4	50	1.3 (7)
Callicarpa subpubescens	13	0.4	7	0.3 (10)
Machilus boninensis	7	0.3	13	0.2 (13)
Melicope grisea var. *grisea*	7	0.1	17	0.2 (12)
Ficus iidana	3	0.0	7	0.1 (14)
Cyathea spinulosa	3	0.0	-	-
Claoxylon centinarium	3	0.0	-	-
Total	1250	82.6	1737	93.5
