# Supplementary material for: Outstanding performance of an invasive alien tree Bischofia javanica relative to native tree species and implications for management of insular primary forests
Source: PeerJ. 2020 Jul 23;8:e9573. doi: 10.7717/peerj.9573 (PMC7382941; doi:10.7717/peerj.9573)
Supplement: Table S2 — The data is from the Chichi-jima Weather Station (Japan Meteorological Agency, 2018). [file peerj-08-9573-s003.pdf]

Table S2 Typhoons for which a wind velocity of more than 20 m s^{-1} was recorded in the Ogasawara Islands between 1987 and 2006. The data is from the Chichi-jima Weather Station (Japan Meteorological Agency, 2018).

Year	Month	Day	Typhoon No.	Name of Typhoon	Min. atmospheric Pressure (hPk)	Mean wind Velocity (m s^{-1})	Max. wind Velocity (m s^{-1})
1987	9	15	13	Freda	986.3	15.5	21.9
1989	10	7	25	Colleen	994.1	13.8	24.4
1991	8	13	10	Ellie	999.5	4.0	21.4
1997	6	13	6	Nestor	999.2	11.7	30.2
1997	9	18	20	David	982.5	12.0	24.9
1997	10	22	24	Joan	994.1	11.5	31.8
1997	11	7	25	Keith	1004.2	10.7	21.5
2002	7	24	9	Fengshen	986.9	11.8	24.6
2002	11	24	25	Haishen	992.2	6.6	20.8
2003	9	29	16	Koppu	983.9	13.8	31.0
2006	9	23	14	Yagi	992.7	14.0	31.5
2006	10	15	18	Soulik	985.6	13.8	25.7
